# Incidence of Veno-Occlusive Disease in Patients with Neuroblastoma Treated with Autologous Stem Cell Transplantation After Chemoimmunotherapy with Temozolomide, Irinotecan, and Dinutuximab Beta

**DOI:** 10.3390/cancers18182912

**Published:** 2026-09-09

**Authors:** Urszula Żebrowska, Walentyna Balwierz, Jolanta Goździk, Aleksandra Krasowska-Kwiecień, Agnieszka Dłużniewska, Monika Richert-Przygońska, Krzysztof Czyżewski, Robert Dębski, Monika Pogorzała, Jan Styczyński, Marek Ussowicz, Agnieszka Sokół, Krzysztof Kałwak, Iwona Malinowska, Michał Romiszewski, Hanna Mańko-Glińska, Wanda Badowska, Tomasz Brzeski, Iwona Świerczek-Skwierawska, Szymon Skoczeń, Aleksandra Wieczorek

**Affiliations:** 1 Department of Pediatric Oncology and Hematology, University Children’s Hospital of Krakow, 30-663 Krakow, Poland; urszula.zebrowska@uj.edu.pl (U.Ż.);; 2 Department of Pediatric Oncology and Hematology, Jagiellonian University Medical College, Wielicka Street 265, 30-663 Krakow, Poland; 3 Jagiellonian University Medical College, Center of Transplantation University Children’s Hospital of Krakow, 30-663 Krakow, Poland; 4 Department of Pediatric Hematology and Oncology, Collegium Medicum, Nicolaus Copernicus University in Torun, Antoni Jurasz University Hospital, 85-094 Bydgoszcz, Polandk.czyzewski@cm.umk.pl (K.C.);; 5 Department of Pediatric Bone Marrow Transplantation, Oncology and Haematology, Wroclaw Medical University, 50-556 Wroclaw, Poland; 6 Department of Oncology, Hematology, Bone Marrow Transplantation and Pediatrics, Medical Universit of Warsaw, 02-091 Warsaw, Polandmichal.romiszewski@uckwum.pl (M.R.); 7 Department of Pediatric Oncology and Hematology, Collegium Medicum, University of Warmia and Mazury in Olsztyn, Regional Specialized Children’s Hospital in Olsztyn, 10-561 Olsztyn, Poland

**Keywords:** neuroblastoma, dinutuximab beta, chemoimmunotherapy, veno-occlusive disease, autologous stem cell transplant, megachemotherapy, BuMel, TreoMel, Busulfan, Treosulfan

## Abstract

**BACKGROUND**: Chemoimmunotherapy (ChIt) has been proven to be an effective treatment for patients with refractory high-risk neuroblastoma (HR-NBL). The objective was to assess the appearance of veno-occlusive disease/sinusoidal obstruction syndrome (VOD/SOS) after high-dose chemotherapy (HDC) and autologous stem cell transplant (ASCT) in patients previously treated with ChIt for refractory HR-NBL. **METHODS**: Nineteen patients were treated with ChIt based on dinutuximab beta for refractory NBL and underwent ASCT. Six patients underwent HDC with ASCT according to the SIOPEN protocol (Busulfan and Melphalan (BuMel)). Treosulfan and Melphalan (TreoMel) were used in 13 patients. The occurrence of VOD/SOS was analysed. The Mann-Whitney U test was used to analyse the correlation between VOD/SOS and potential risk factors. **RESULTS**: VOD/SOS developed in five out of six patients when BuMel was used after ChIt (3 pts, grade 4; 2 pts, grade 3), resulting in two deaths. No VOD/SOS was observed in patients receiving TreoMel. Other analysed factors had no influence of VOD/SOS occurrence and the only identified risk factor was BuMel administered after ChIt. **CONCLUSIONS**: Patients with refractory HR-NBL treated with ChIt and DB did not present life-threatening toxicities or severe liver toxicities during the ChIt cycles and continued to HDC with ASCT according to SIOPEN guidelines. The incidence of severe VOD/SOS after ChIt and subsequent BuMel was higher than expected, with severe course resulting in two deaths. Taking into account the possible increase in the risk of VOD/SOS after BuMel used after chemotherapy combined with DB, careful observation with early VOD/SOS treatment in patients after ChIt is recommended and TreoMel regimen may be considered as less toxic.

## Simple Summary

Patients with high-risk neuroblastoma (HR-NBL) who do not respond sufficiently to treatment are considered refractory. These patients have poor prognosis with conventional therapy; however, they show a good response to a combination of chemotherapy and immunotherapy with dinutuximab beta. The aim of the study was to assess the occurrence of veno-occlusive disease/sinusoidal obstruction syndrome (VOD/SOS) after high-dose chemotherapy and autologous stem cell transplant in patients previously treated with chemoimmunotherapy (ChIt) for refractory HR-NBL. The only risk factor for severe VOD/SOS identified in the study was Busulfan and Melphalan (BuMel); however, the risk of severe VOD/SOS after BuMel seems higher in patients previously treated with ChIt. Due to the increasing use of ChIt in refractory HR-NBL and future perspectives for first-line treatment, appropriate observation with early VOD/SOS treatment and consideration of TreoMel as a potentially safer high-dose chemotherapy regimen may be advisable.

## 1. Introduction

Neuroblastoma (NBL) is one of the most common causes of death from childhood cancer [1]. The addition of antibodies directed against the disialoganglioside GD2 antigen (aGD2) in front-line maintenance therapy in high-risk disease (HR) improved survival [2,3], but still 10–20% of patients never achieve remission and are considered refractory to treatment, while another 50% experience a relapse [4].

Treatment options for refractory NBL involve intensive multimodal approaches [5,6]. One of the treatment modalities with proven effect on the increase in response rates is chemoimmunotherapy (ChIt), based mainly on temozolomide and irinotecan or topotecan. The synergy of cytostatic drugs with dinutuximab beta (DB) was shown in in vivo models [7], and the direct cytotoxic effect of aGD2 antibodies was shown in NBL cell lines [8]. The results of the BEACON study show an overall response rate (ORR) of 35% for ChIt and 18% for chemotherapy only [9]. There is also real-life data on chemotherapy in combination with DB, with the ORR in relapse/refractory (R/R) cohort over 60% [10,11]. The role of ChIt in relapsed patients was also shown in the SACHA study [12]. More intensive chemotherapy was also reported, with a response rate of 48% [13]. Promising data were reported for hu14.18K322A with standard induction chemotherapy, which showed 73.7% (95% CI, 60.0 to 83.4) 3-year event-free survival (EFS) [14]. Children who achieve the sufficient response proceed to the consolidation phase. High-dose chemotherapy (HDC) and autologous stem cell transplant (ASCT) is the standard of care in Children’s Oncology Group (COG) and International Society of Paediatric Oncology Europe Neuroblastoma (SIOPEN) protocols and has a proven impact on treatment outcome [15]. It remains the treatment of choice, with efficacy confirmed in randomised clinical trials [16,17,18]. COG developed carboplatin, etoposide, and melphalan (CEM) as HDC regimen. SIOPEN recommends using busulfan and melphalan (BuMel) as HDC regimen, which was more effective and safer than CEM in randomised clinical trial [15]. While the frequency of infection, fever, and renal toxicity was higher in patients in the CEM arm, the rate of reversible veno-occlusive disease/sinusoidal obstruction syndrome (VOD/SOS) was higher in the BuMel arm (22% vs. 9%) [15]. VOD/SOS is a potentially fatal complication of ASCT, recognised as transplant-related systemic endothelial diseases [19]. Busulfan may cause damage to the endothelium of small vessels, which leads to the formation of microthrombosis. The entire process results in hypertension in the portal vessels and life-threatening complications. The incidence of severe VOD/SOS in different studies ranged from 4% to 26% [20,21,22]. Grade 3 liver toxicities included ascites, hyperbilirubinemia, liver pain, hypoxia, and weight gain [13]. The objective of this report was to evaluate the incidence of VOD/SOS after HDC in patients treated previously with ChIt for therapy of refractory HR-NBL.

## 2. Materials and Methods

From June 2019 to June 2026, 79 patients with HR-NBL received chemoimmunotherapy (55 patients with relapse disease, 22 patients with refractory disease, 1 patient treated with chemoimmunotherapy as maintenance treatment). Retrospective review of medical records was performed of all 19 patients with refractory HR-NBL who underwent HDC with ASCT after ChIt in Poland.

All patients after ASCT were regularly monitored for signs of the VOD/SOS, according to the standards of Joint Accreditation Committee—ISCT and EBMT [19,23,24,25,26,27,28,29,30]. Patients who developed VOD/SOS were subjected to this analysis, according to the diagnostic criteria and severity levels developed by the European Society for Blood and Marrow Transplantation (EBMT) [19,20]. “At least two of the following criteria are necessary to make the diagnosis of VOD/SOS: unexpected and refractory thrombocytopenia, unexplained weight gain, hepatomegaly, ascites, increase in bilirubin concentration on 3 consecutive days or bilirubin ≥2 mg/dL within 72 h” (details are presented in Additional file, Table S1) [19]. EBMT distinguished four stages of severity of VOD/SOS: mild, moderate, severe, and very severe [19].

Retrospective analysis of data was approved by the Research Ethics Committee of the Jagiellonian University Medical College in Krakow, Poland (118.0043.1.101.2024 from 19 April 2024).

### 2.1. Patients and Treatment

The diagnosis of HR-NBL was based on the International Neuroblastoma Risk Group (INRG) Staging System. All patients were initially treated according to the HR-NBL-SIOPEN-1 protocol, with COJEC induction chemotherapy. Patients were considered refractory if, after induction, they did not achieve remission sufficient to proceed to HDC as defined in the SIOPEN protocol [15]. The additional chemotherapy before ChIt was allowed according to the decision of treating physicians.

All patients received ChIt based on DB administered as a continuous infusion at 10 mg/m^2^/day during 5 days, on days 2 to 6 of each 21 day cycle. All patients received the standard recommended supportive treatment [31].

The first choice chemotherapy was temozolomide and irinotecan (TemIri) for five days (treatment details are presented in Additional file, Table S2), administered in 17 of the 19 patients analysed. Thirteen out of seventeen patients continued the entire therapy with the TemIri without any modifications. One patient received only 3 cycles of TemIri due to disease progression in the central nervous system. He was transplanted in complete response (CR) after additional conventional chemotherapy and he was included in the analysis. In 4 patients, change in chemotherapy was necessary—1 patient had irinotecan changed to topotecan and, in 3 patients, GPOH chemotherapy was administered due to disease progression [13,32]. Two of nineteen patients with refractory disease received two cycles of GPOH therapy as the first choice (Additional file, Table S3) and, after two cycles, it was changed for TemIri [10,13].

The number of cycles was not defined before the start of treatment and it was dependent on tolerance, toxicity, and response to treatment. Patients received between 3 and 11 cycles.

After achieving sufficient response, the patients with previous refractory disease continued therapy according to the SIOPEN guidelines, proceeding to HDC with ASCT. According to the decisions of the clinicians and the guidelines of the VERITAS protocol [33], six patients (31.6%) received ^131^I-MIBG therapy before HDC, and one patient (5.6%) with persistent non-avid MIBG disease had tandem transplantation with thiotepa [33] (Table 1). The HDC of choice was BuMel, as recommended for first-line therapy in the SIOPEN protocol. After the observation of serious VOD/SOS in the first patients, HDC based on Treosulfan and Melphalan (TreoMel) was the treatment option, left to the decision of the transplant centres (Table 2).

### 2.2. Assessments and Outcomes

The disease was evaluated after COJEC chemotherapy and before ChIt, if additional treatment was administered (Additional file, Table S4). Response was evaluated after cycle 3, at the end of treatment (5 or 7 cycles, depending on the response after 3 cycles), and when progression/relapse was suspected. The best response rate was 13 PR and 6 CR assessed based on International Neuroblastoma Response Criteria [35] (Table 1).

Adverse events were classified according to the Common Terminology Criteria for Adverse Events (CTCAE) version 4.0. Pain was evaluated using the Wong–Baker FACES Pain Rating Scale and the Face, Legs, Activity, Cry, Consolability (FLACC) scale in patients under three years of age, with both scales ranging from 0 (no pain) to 10 (worst pain imaginable) [6].

### 2.3. Statistical Analysis

The data cutoff was 30 June 2026.

Safety data were analysed for all patients according to the type of chemotherapy used during ChIt and the type of HDC. The Mann–Whitney U test was used to statistically analyse the correlation between ferritin levels, previous liver disturbances, time from ChIt to transplantation, and the number of cycles with temozolomide, irinotecan, and the higher risk of VOD/SOS.

## 3. Results

In this study, 19 patients diagnosed with refractory HR-NBL undergoing ChIt followed by HDC with ASCT were analysed. All patients presented with INRG stage M NBL at diagnosis. There were 9 girls (47.4%) and 10 boys (52.6%), with a median age of 30.7 months (IQR 19,75–41,17). MYCN amplification was found in 7 patients (36.8%), and ALK mutation in 3 (15.8%) out of 19 patients (Additional file, Table S5).

### 3.1. Treatment and Outcome

After completion of ChIt, all patients with sufficient response were qualified for HDC with ASCT. The choice of therapy prior to autoSCT (single vs. tandem HDC or ^131^I-MIBG therapy) was based on treatment response. Patients with persistent metastases were qualified for tandem HDC (thiotepa) if the lesions were non-MIBG-avid (*n* = 1) or for ^131^I-MIBG therapy if lesions were MIBG-avid (*n* = 6). A total of 6 out of 19 patients received BuMel according to the SIOPEN protocol and 13 received TreoMel. Two out of six patients who received BuMel received MIBG therapy prior to HDC and one patient received Thiotepa. Four out of thirteen patients who received TreoMel received MIBG therapy prior to HDC. By the clinicians’ decision, one patient with PR and non-MIBG-avid lesions was qualified for single HDC with BuMel. The time from the end of ChIt to transplantation ranged from 1 to 3 months in patients without disease progression and 4 months in a patient who required additional cycles of chemotherapy. Three patients in whom the ALK mutation was found were treated with lorlatinib before, but not during HDC.

### 3.2. Toxicity of Chemoimmunotherapy

ChIt was well tolerated in all patients. No severe liver or lung toxicities were observed during temozolomide- or GPOH-based ChIt. None of the patients with refractory NBL developed VOD/SOS.

### 3.3. Toxicity of HDC

All patients developed adverse events commonly associated with HDC. Five patients (26.3%) out of nineteen developed VOD/SOS (grade 3, *n* = 2; grade 4, *n* = 3). All of them received BuMel, one patient received additionally Thiotepa as the tandem HDC. Two patients underwent previously ^131^I-MIBG therapy and one of them also received lorlatinib. Patients who did not develop VOD/SOS (*n* = 14) received BuMel (*n* = 1) or TreoMel (*n* = 13). Three out of thirteen patients (23.1%) who received TreoMel underwent ^131^I-MIBG therapy and one received ^131^I-MIBG therapy and lorlatinib. Two patients receiving BuMel died from VOD/SOS. One patient with VOD/SOS grade 3 died 4.47 months after transplantation due to further disease progression in the central nervous system (CNS). Nine patients are alive in CR (7 after TreoMel and 2 after BuMel HDC), with the follow-up being 11.30–56.14 months (median 41.72, IQR 27.28–47.09) from the beginning of ChIt and 6.37–53.71 months (median 35.40, IQR 22.91–39.51) from HDC. Two patients with PR were still treated at the end of the follow-up period. Three out of thirteen patients (23.1%) who received TreoMel had disease progression and remain on treatment. Two patients out of thirteen (15.38%) relapsed after TreoMel (14.29 months after ChIt and 9.69 months after HDC 15.51 months after ChIt and 8.05 months after HDC) and died due to further disease progression.

### 3.4. VOD/SOS

The time of development of VOD/SOS symptoms was similar for the four patients, with first symptoms about 3 weeks after transplantation (16–18 days). One patient developed VOD/SOS 36 days after ASCT and the course of the disease was rapid, with death less than 24 h after admission to the intensive care unit. All patients who developed VOD/SOS met the criteria of EBMT for hepatic VOD/SOS in children (Table 3) [19].

Three of them had VOD/SOS grade 4 and two VOD/SOS grade 3 [19]. The patients presented with characteristic symptoms of VOD/SOS, such as increased bilirubin (3/5), hepatomegaly (4/5), increased liver enzymes (5/5), reverse flow in hepatic vessels (3/5), thrombocytopenia (5/5), coagulation disorders (4/5), and ascites (5/5) (Table 4). Additionally, fluid in pleural cavities and oesophageal varices were observed in one patient. In one patient, signs of developing collateral circulation were found.

Prophylaxis against liver VOD/SOS (such as ursodeoxycholic acid and antithrombin III) were used in one patient with resistant thrombocytopenia before other clinical symptoms occurred, but it did not prevent grade 4 VOD/SOS. No other patients received defibrotide in prophylaxis, as this is not reimbursed as standard of care in Poland. All patients with VOD/SOS symptoms received treatment according to the guidelines (Additional file, Table S6).

All patients with VOD/SOS symptoms received therapy with defibrotide at the standard dose for 4 to 23 days. One patient received defibrotide early after the occurrence of thrombocytopenia without any other symptoms of VOD/SOS and, in spite of this, the patient developed grade 4VOD/SOS.

Four out of five patients with severe VOD/SOS received methylprednisolone intravenously and one of them continued oral steroid therapy with prednisone for 3 months with gradual dose reduction. In two of them, high-dose steroid therapy (500 mg/m2 every 12 h) was started at an early stage of VOD/SOS (on days 2 and 3 of VOD/SOS, respectively). Both patients with early steroid therapy survived.

Supportive treatment included correction of coagulation disorders by plasma transfusions (*n* = 2), cryoprecipitate (*n* = 1), antithrombin III (*n* = 4), and vitamin K (*n* = 5). Fluid restriction, stimulation of diuresis with furosemide and albumin infusion, and/or spironolactone were used in all five patients.

In two cases, grade 4 VOD/SOS led to the death of patients despite adequate supportive therapy and the use of defibrotide, on the first and third day from the beginning of symptoms. Death occurred on days +41 and +43 after transplantation and 24 and 4 days after the beginning of VOD/SOS symptoms. The remaining three patients (with VOD/SOS grade 4 (*n* = 1) and grade 3 (*n* = 2)) recovered and were able to continue further treatment (maintenance with DB) without serious side effects.

In two patients with severe VOD/SOS, disease progression was observed. One of them died due to CNS metastases, while the second started the third-line therapy. The third patient, who recovered after grade 4 VOD/SOS, was able to undergo radiotherapy and immunotherapy with DB. The patient finished the planned therapy in CR, without the recurrence of VOD/SOS or any severe liver toxicities.

None of the patients who received TreoMel (*n* = 13) developed VOD/SOS. In addition to the typical post-transplant complications, acute pancreatitis, enteritis, elevated transaminases, and urinary tract infection were observed in one patient, and sepsis occurred in another patient. These patients were qualified for subsequent treatment according to the HR-NBL-SIOPEN protocol. All of them underwent local radiotherapy and received maintenance DB according to the protocol. Two of them did not present severe side effects during maintenance therapy. In one patient, immunotherapy was permanently discontinued due to severe capillary leak syndrome (CTCAE grade 4).

One patient received ChIt and underwent BuMel HDC with ASCT without developing VOD/SOS. The patient has completed treatment and remains in CR.

Potential risk factors of VOD/SOS were analysed. Time between ChIt and HDC was within the range of 26–103 days (MEDIAN = 45, IQR 34–74, *p* = 0.920). The number of chemotherapy cycles (including treatment before ChIt) with temozolomide was in the range from 3 to 19 (MEDIAN = 6, IQR 5–6). The number of cycles with irinotecan was in the range from 3 to 7 (MEDIAN = 5; IQR 3–6). There was no statistical significance between the number of chemotherapy cycles and occurrence of VOD/SOS (*p* = 0.1627, *p* = 0.084, respectively). There was no statistically significant correlation between ferritin concentration and the risk of VOD/SOS (*p* = 0.462). The values ranged from 221 ug/L to 2625 ug/L (MEDIAN = 604.5, IQR 259,5–756,6).

## 4. Discussion

Chemoimmunotherapy with the use of anti-GD2 antibodies and different chemotherapy schedules has become the treatment of choice for relapsed and refractory NBL with a relatively higher response rate than chemotherapy alone [2,3,4,10,11]. The synergy of both methods was proved in spheroid models, where their combination showed superior efficacy compared with chemotherapy or DB alone [7]. The promising results were also shown for humanised anti-disialoganglioside monoclonal antibody (hu14.18K322A) used in induction, with 97% of patients achieving at least PR at the end of induction, with an acceptable toxicity profile [14].

Although there are different attitudes concerning consolidation therapy, both SIOPEN and COG recommend it as the standard therapy both in first-line and refractory patients and as an option in relapsed patients [36,37]. High-dose therapy, although its positive impact on EFS is well proven [16,17,18], is associated with toxicities. The main concern of recommended European HDC, consisting of Busulfan and Melphalan, is the occurrence of VOD/SOS. The overall rate of VOD/SOS after BuMel is 5.9% (ANBL12P1) to 22% (SIOPEN) and severe VOD/SOS 4% in both cohorts [15,21]. The difference may be caused by the different induction schedules and the different criteria used to define VOD/SOS in both studies.

Most publications concerning ChIt either do not report the further treatment or, if it is reported, the data concerning toxicities of the consecutive therapies are not included. Some data are reported in Furman et al., in which BuMel was used in combination with parental NK cells and hu14.18K322A. Two patients who received hu14.18K322A developed a hemophagocytic lymphohistiocytosis-like syndrome; so, the drug was eliminated from this treatment phase. One patient died during ASCT, but no reasons of death are reported [14]. There are no data reported on toxicities or potential prophylaxis of VOD/SOS. In the SACHA study, five patients received tandem transplantation, but there are no data concerning the type of HDC and toxicities [12]. In the study of Flaadt et al. concerning haplotransplantation, there was no occurrence of grade 4 VOD/SOS, but there are no data concerning patients after ChIt included in the study and Busulfan was not used in this group [36].

In the presented cohort, we analysed consecutive patients with refractory NBL, who received ChIt and achieved sufficient response to proceed to the consolidation phase. This part of therapy was not standardised. Among the 19 patients who continued to consolidation, BuMel was used alone (*n* = 3) or in combination with thiotepa (*n* = 1) or ^131^I-MIBG (*n* = 2). Five out of six patients developed VOD/SOS; in five patients, criteria of VOD/SOS grade 3 or 4 were met. Three patients with grade 4 VOD/SOS received very intensive treatment including defibrotide, but despite it, two patients died from this complication. For this reason, the choice of HDC for the next patients was left to the decision of the treatment centres, and HDC based on TreoMel was administered in 13 patients, without severe toxicities. Treosulfan is not a drug of choice in HDC in HR-NBL, but there is some evidence that treosulfan-based HDC may be safe and effective in this group of patients [34,38,39].

Most cases of VOD/SOS occurred about 17 days after stem cell infusions and were characterised by severe liver disturbance. Analysis of possible predisposing factors (previous hepatotoxicity, ferritin level, time between ending ChIt and HDC, number of cycles with temozolomide or irinotecan) did not show any differences between patients receiving Busulfan or treosulfan [40,41]. Hepatotoxic chemotherapy may be a risk factor for VOD/SOS after HDC. Temozolomide has confirmed hepatotoxicity, but in the available data, there is no clear correlation between temozolomide and HDC toxicities [42,43]. However, data on temozolomide with consecutive ASCT are scarce and come primarily from brain tumour therapy; so, temozolomide therapy cannot be excluded as a potential risk factor for VOD/SOS [42]. In our cohort (own unpublished data), two patients with relapsed NBL, treated according to the GPOH protocol with DB, developed symptoms of VOD/SOS, presenting with ascites, coagulation disorders, hepatomegaly, and elevated transaminase and bilirubin levels. Both patients required peritoneal drainage and were treated with defibrotide. One patient had previously undergone HDC followed by autologous stem cell transplantation, whereas the other had previously undergone both ASCT and allotransplantation. No VOD/SOS was observed in patients treated with the temozolomide-based protocol.

In adult patients with liver cancer metastases, VOD/SOS may occur in patients who receive alkylating agents. This was observed for oxaliplatin, but irinotecan did not increase the risk of VOD/SOS [44]. Drug-induced liver injury (DILI) as a possible complication of cytostatic drugs, including alkylating agents, and idiosyncratic DILI can develop after a variable period of latency, especially if drugs are used in combination with new agents [45,46]. Furthermore, in some reported patients, an additional therapy was administered as part of consolidation, which could also influence the risk of hepatotoxicity, although we did not observe statistical significance. Two patients with VOD/SOS received ^131^I-MIBG therapy before BuMel according to the VERITAS protocol guidelines [33], but according to the available data, it should not increase the risk of VOD/SOS [47]. Moreover, ^131^I-MIBG therapy was used in both the BuMel and TreoMel groups. One patient with VOD/SOS received thiotepa, which can also be a risk factor for VOD/SOS, but mainly in patients after radiotherapy [30]. The potential role of DB therapy as a risk factor for VOD/SOS is unclear. The preceding induction of inflammation might be a predisposing factor to hepatotoxicity after HDC. The Fc part of different monoclonal antibodies (mAbs) can bind to hepatocytes and to the liver sinusoidal endothelial cells (LSECs), which plays a role in different immunological phenomena including the ones involved in mAbs therapy. Vascular toxicity caused by immune complex deposition and complement-mediated toxicity toward the LSEC may present as VOD/SOS [48,49]. There is a higher risk of VOD/SOS reported for gemtuzumab ozogamicin and inotuzumab ozogamicin [50,51]. Sporadic cases of severe hepatotoxicity have also been described for other immunotherapy agents, like check-point inhibitors and immunomodulators [52]. Despite the small sample size of the analysed cohort, the type of HDC used after ChT was the only risk factor for VOD/SOS that we identified. However, the small number of patients represents an important limitation of our study, as it may affect the generalizability of our findings and makes it difficult to draw definitive conclusions. Therefore, these results should be interpreted with caution. Another important issue is the severity of VOD/SOS and the deaths associated with this complication, which highlights the importance of reporting our findings despite the small study population [26]. Although the TreoMel schedule has no proven efficacy in NBL, there is evidence suggesting its potential effectiveness and, in patients treated with ChIt, this regimen may be considered a preferable therapeutic option [34].

## 5. Conclusions

The causal correlation of chemoimmunotherapy and the risk of severe VOD/SOS after Busulfan, leading to death, cannot be definitely proved on the basis of the reported case series. Taking into account the risk of severe, life-threatening VOD/SOS potentially associated with the use of Busulfan after previous administration of ChIt, in view of the increasing successful use of ChIt in patients with R/R NBL and future perspectives of its use in the first-line setting, special strict monitoring of this group of patients or optional early therapy, such as high-dose steroids, or defibrotide prophylaxis should be considered. TreoMel could be considered as the alternative to BuMel to enable consolidation after effective induction with chemotherapy and dinutuximab beta. However, its efficacy has to be confirmed in clinical trials.

## 6. Limitations

The limitation of the study is its retrospective character, the heterogeneous therapy administered before HDC, and the low number of patients. The potential causal relationship between chemoimmunotherapy and HDC with Busulfan should be evaluated in preclinical studies and further clinical observations.

## Figures and Tables

**Table 1:** Disease status before ASCT, type of ASCT, and response at the end of follow-up.

Patient No	Best Response for ChIt	Status of the Disease Before ASCT	^131^I-MIBG Therapy	Type of HDC	Status of the Disease in ^131^I-MIBG Before ASCT	Response at the End of Follow-Up
1	PR	PR	Yes	BuMel	MIBG avid disease	death due to VOD/SOS
2	PR	PR	Yes	BuMel	MIBG avid disease	CR
3	PR	PR	No	Thiotepa + BuMel	Non-avid MIBG	death due to VOD/SOS
4 *	PR	PR	No	BuMel	Non-avid MIBG	death due to PD
5	PR	PR	Yes	TreoMel	MIBG avid disease	CR
6	PR	PR	Yes	TreoMel	MIBG avid disease	CR
7	CR	CR	No	TreoMel	CR	PD
8	CR	CR	No	BuMel	CR	death due to PD
9	CR	CR	No	BuMel	CR	CR
10	PR	PR	Yes	TreoMel	MIBG avid disease	CR
11	CR	CR	No	TreoMel	CR	CR
12	CR	CR	No	TreoMel	CR	PD
13	CR	CR	No	TreoMel	CR	PD
14	PR	PR	No	TreoMel	CR	death due to PD
15	PR	PR	No	TreoMel	CR	CR
16	PR	PR	No	TreoMel	CR	CR
17	PR	PR	Yes	TreoMel	MIBG avid disease	PR
18	PR	PR	No	TreoMel	CR	PR
19	PR	PR	No	TreoMel	CR	CR

PR—partial response; CR—complete response; ^131^I-MIBG—131 iodine meta-iodobenzylguanidine; ASCT—autologous stem cell transplant; BuMel—Busulfan i.v., Melphalan; TreoMel—Treosulfan; Melphalan; ChIt—chemoimmunotherapy; PD—progression disease; VOD/SOS—veno-occlusive disease/sinusoidal obstruction syndrome. * Despite PR, the patient was referred to the single HDC by the clinicians’ decision.

**Table 2:** Protocols for high-dose chemotherapy [15,21,34].

HDC	Drugs	Dose	Days of Administration
BuMel	Busulfan i.v.	Dose per kg according to SIOPEN protocol	−6 to −3
	Melphalan i.v.	140 mg/m^2^/day	−1
TreoMel	Treosulfan i.v.	10 g/m^2^	−8 to −6
	Melphalan i.v.	70 mg/m^2^	−2 to −1

HDC—high-dose chemotherapy; BuMel—Busulfan, Melphalan; TreoMel—Treosulfan, Melphalan.

**Table 3:** Occurrence of the EBMT criteria for hepatic VOD/SOS in analysed patients.

Patient	Thrombocytopenia (Day After Transplantation)	Weight Gain	Hepatomegaly	Ascites	Increase in Bilirubin (Day After Transplantation)	VOD/SOS Grade
1	+36	yes	yes	yes	+38	4
2	+18	yes	yes	yes	+18	4
3	+17	yes	yes	yes	+17	4
4	+16	yes	yes	yes	no	3
5	+17	yes	no	yes	no	3

**Table 4:** Severity of symptoms of VOD/SOS according to CTCAE 4.0.

Patient	Bilirubin	ALT	AST	GGT	Thrombocytopenia	APTT	Decreased ATIII	Decreased Fibrinogen	Increased D-Dimers
1	3	2	3	1	4	1	yes	no	no
2	1	2	2	3	4	3	yes	yes	yes
3	4	4	4	2	3	1	yes	no	yes
4	-	3	4	-	4	3	no	no	yes
5	-	1	1	2	4	no	no	yes	yes

ALT—alanine transaminase; AST—aspartate transaminase; APTT—activated partial thromboplastin time; GGT—gamma-glutamyltransferase; CTCAE—Common Terminology Criteria for Adverse Events; ATIII—antithrombin III.

## Data Availability

Data used for this paper were collected from the electronic medical records of the patients included in the study and are not publicly available due to general data protection regulations. The anonymised data presented in this study are available upon request from the academic sponsor.

## Supplementary Materials

The following supporting information can be downloaded at: https://www.mdpi.com/article/10.3390/cancers18182912/s1, Table S1: Diagnostic criteria of VOD/SOS according to the European Society for Blood and Marrow Transplantation [19]; Table S2: Schedules of chemoimmunotherapies – ToTem and TemIri; Table S3: The schedules of chemoimmunotherapies - GPOH; Table S4: Patient status and treatment used before and during chemoimmunotherapy; Table S5: Demographic data and disease characteristic; Table S6: Treatment during VOD/SOS.

## Abbreviations

The following abbreviations are used in this manuscript:

^131^I-MIBG	131 iodine meta-iodobenzylguanidine
ALT	Alanine transaminase
APTT	Activated partial thromboplastin time
ASCT	Autologous stem cell transplant
AST	Aspartate transaminase
ATIII	Antithrombin III
BM	Bone marrow
BuMel	Busulfan, Melphalan
CARBO	Carboplatin
CADO	Cyclophosphamide, doxorubicin, vincristine
CEM	Carboplatin, etoposide, melphalan
c.i.	Continuous infusion
ChIt	Chemoimmunotherapy
CNS	Central nervous system
COG	Children’s Oncology Group
COJEC	Cisplatin, vincristine, carboplatin, etoposide, cyclophosphamide
CR	Complete response
CT	Computer tomography
CTCAE	Common Terminology Criteria for Adverse Events
DB	Dinutuximab beta
DILI	Drug-indicated liver injury
EBMT	European Society for Blood and Marrow Transplantation
EFS	Event-free survival
FLACC	The Face, Legs, Activity, Cry, Consolability
GGT	Gamma-glutamyltransferase
GM-CSF	Granulocyte macrophage colony-stimulating factor
GPOH	Gesellschaft fur Pädiatrische Onkologie und Hämatologie/Society for Pediatric Oncology and Hematology
HDC	High-dose chemotherapy
HR	High-risk
i.v.	Intravenously
LINES	European Low and Intermediate Risk Neuroblastoma Protocol
LSEC	Liver sinusoidal endothelial cells
mAbs	Monoclonal antibodies
MRI	Magnetic resonance imaging
NBL	Neuroblastoma
NCA	Numerical chromosomal aberrations
ORR	Overall response rate
OS	Overall survival
PD	Progression disease
p.o.	Orally
PR	Partial response
Pts	Patients
R/R	Relapsed/refractory
RR	Response rate
SCA	Structural chromosomal aberrations
SD	Stable disease
SIOPEN	International Society of Paediatric Oncology Europe Neuroblastoma
SOS	Sinusoidal obstruction syndrome
TemIri	Temozolomide, Irinotecan
ToTem	Temozolomide, Topotecan
TopoCyclo	Topotecan, Cyclophosphamide
TreoMel	Treosulfan, Melphalan
VOD	Veno-occlusive disease
VP	Etoposide
