# Supplementary material for: Incidence of Veno-Occlusive Disease in Patients with Neuroblastoma Treated with Autologous Stem Cell Transplantation After Chemoimmunotherapy with Temozolomide, Irinotecan, and Dinutuximab Beta
Source: Cancers (Basel). 2026 Sep 9;18(18):2912. doi: 10.3390/cancers18182912 (PMC13604533; doi:10.3390/cancers18182912)
Supplement: Supplementary file 1 [file cancers-18-02912-s001.zip › cancers-4506010-supplementary.pdf]

Supplementary Table S1 Diagnostic criteria of VOD/SOS according to the European Society for Blood and Marrow Transplantation [19]

No time limit for the onset of VOD/SOS after ASCT
Presence of **at least two** of the following symptomps:
• Unexplained thrombocytopenia refractory to platelet transfusions.
• Unexplained weight gain over 3 consecutive days despite the use of diuretics, or weight gain exceeding 5% of baseline body weight.
• Hepatomegaly (ideally confirmed by imaging) exceeding the patient's baseline liver size.
• Ascites (ideally confirmed by imaging) exceeding the patient's baseline amount of free fluid in the peritoneal cavity.
• An increase in bilirubin levels from baseline over 3 consecutive days, or a bilirubin level of at least ≥2 mg% (≥34mmol/l).

Supplementary Table S2 Schedules of chemoimmunotherapies – ToTem and TemIri

Type of chemoimmunotherapy	Drug	Dose	Days of administration
TemIri + DB	Temozolomide	100 mg/m^2/day p.o.	1-5
	Irinotecan	50 mg/m^2/day i.v.	1-5
	Dinutuximab beta	10 mg/m^2/day c.i.	2-6
ToTem + DB	Temozolomide	100 mg/m^2/day p.o.	1-5
	Topotecan	0,75 mg/m^2/day i.v.	1-5
	Dinutuximab beta	10 mg/m^2/day c.i.	2-6

ToTem - Temozolomide, Topotecan, TemIri - Temozolomide, Irinotecan, DB - dinutuximab beta, p.o. – orally, i.v. – intravenously, c.i – continuous infusion

Supplementary Table S3 The schedules of chemoimmunotherapies - GPOH

GPOH Protocol + DB			
Type of chemoimmunotherapy	Drug	Dose	Days of administration
Cycle N5 + DB	Vindesine	3 mg/m²/day i.v.	1
	Cisplatin	40 mg/m²/day c.i.	1-4
	Etoposide	100 mg/m²/day c.i.	1-4
	Dinutuximab beta	10 mg/m²/day c.i.	5-10
Cycle N6 + DB	Vincristine	1,5 mg/m²/day i.v.	1 and 8
	Dacarbazine	200 mg/m²/day i.v.	1-5
	Ifosfamide	1500 mg/m²/day c.i.	1-5
	Doxorubicin	300 mg/m²/day i.v.	6 and 7
	Dinutuximab beta	10mg/m²/day c.i.	6-10

GPOH – German Society of Paediatric Oncology and Haematology, DB - dinutuximab beta; i.v. – intravenously, c.i – continuous infusion

Supplementary Table S4 Patient status and treatment used before and during chemoimmunotherapy

Patient No	Treatment before ChIt	Status of the disease before ChIt	Type of ChIt	Cycles of ChIt	Other treatment	Liver metastases
1	2 cycles of VP+CARBO,<br>3 cycles of CADO (LINES group 10),<br>13 cycles of Temodal+Topotecan,	No response, MIBG after 5 cycles - primary tumour with liver and bone metastases	TemIri+DB	6	1 cycle N5, 1 cycle N6; ALK inhibitor (5 months, with a break in the ASCT period) PR during ALK inhibitor treatment	yes
2	COJEC,<br>2 cycles of Temodal+Irinotecan	Regression of lymph node lesions in the abdomen and pelvis, persistent metastases in bones of the skull (>3)	TemIri+DB	6	no	no
3	COJEC	Reduction of primary tumour size, increased infiltration of the BM, reduction of three previous bone lesions in MIBG with a new lesion in the vertebra, suspicious nodules in the	TemIri+DB	7	no	no

		lungs.				
4	VP16/CARBO, COJEC,<br>2 cycles of TVD, 2 cycles of TemIri	CR in soft tissue in MIBG scan, no response of multiple bones metastasis	TemIri+DB<br>ToTem+DB	1<br>3	no	yes
5	LINES 10 group, 8 cycles of VP/CARBO; relapse treatment: 4 cycles of TOTEM, COJEC	Persistent brain lesions, progression of metastases, persistent primary tumour (not resectable)	N6+DB<br>N5+DB<br>TemIri+DB	1<br>1<br>2	no	no
6	COJEC, 2 cycles of TVD	Persistent bone metastases	TemIri+DB<br>N6+DB<br>N5+DB<br>TopoCyclo+DB	5<br>1<br>1<br>1	Lorlatinib - CR	no
7	COJEC	Persistent metastases in BM, lymph nodes, primary tumour.	TemIri+DB	5	no	no
8	COJEC	Persistent BM metastases	TemIri+DB	3	2 cycle of ICE (PD)	no
9	COJEC	Persistent bone metastases	TemIri+DB	3	no	no
10	COJEC	Persistent bone metastases	TemIri+DB	8	no	no
11	COJEC	Persistent bone metastases, 2% BM involvement in	TemIri+DB	5	no	no

		trephine				
12	COJEC	Persistent bone metastases	TemIri+DB	5	no	no
13	COJEC	Persistent bone metastases	TemIri+DB	5	no	no
14	COJEC	SD in MIBG scan, massive BM involvement	GPOH+DB<br>TemIri+DB<br>ToTem+DB	2<br>1<br>1	no	no
15	COJEC	Persistent bone metastases	TemIri+DB	5	no	no
16	COJEC	Persistent bone metastases	TemIri+DB	5	Lorlatinib - CR	no
17	COJEC	Persistent bone metastases	TemIri+DB<br>GPOH+DB<br>Topo/Cyclo	7<br>2<br>2	no	no
18	COJEC	PR in primary tumor (no resectable) and liver metastases	TemIri	7	no	yes
19	COJEC	Persistent in BM and bones metastases	TemIri	6	no	no

ChIt - Chemoimmunotherapy; VP - Etoposide, CARBO - Carboplatin, CADO - Cyclophosphamide, Doxorubicin, Vincristine; LINES - European Low and Intermediate Risk Neuroblastoma Protocol; TemIri -Temozolomide, Irinotecan; MIBG - Iodine meta-iodobenzylguanidine; DB - Dinutuximab beta; BM - Bone Marrow; CR -Complete Response, SD - Stable Disease, MRI - Magnetic Resonance Imaging, TOTEM - Topotecan, Temozolomide; TopoCyclo - Topotecan, Cyclophosphamide; COJEC - Cisplatin, Vincristine, Carboplatin, Etoposide, and Cyclophosphamid

Supplementary Table S5 Demographic data and disease characteristic

Characteristics	Patients (N)
Age at diagnosis	
< 18mo	4
≥ 18mo	15
Stage 4	19
MYCN status	
amplified	7
non-amplified	12
ALK mutation	3
NCA	2
SCA	5

Mo – months, NCA - numerical chromosomal aberrations, SCA - structural chromosomal aberrations

Supplementary Table S6 Treatment during VOD/SOS

Grade of VOD/SOS	Applied treatment
Grade 3 (n=2)	defibrotide,<br>methylprednisolone,<br>vitamin K, antithrombin III, platelet transfusions, cyclonamine, ornithine, phospholipids,<br>furosemide, albumin, fluid restriction, spironolactone,
Grade 4 (n=3)	defibrotide,<br>methylprednisolone, prednisone<br>vitamin K, antithrombin III, platelets, plasma transfusions, cryoprecipitate, ornithine, tranexamic acid, timonacic, phospholipids,<br>furosemide, albumin, fluid restriction, spironolactone, dobutamine, mechanical ventilation, dialysis
